# Outcomes of Hartmann’s Surgery in Colonic Diverticulitis

**DOI:** 10.7759/cureus.111425

**Published:** 2026-06-24

**Authors:** Tien Tran Phung Dung, Huan Nguyen Ngoc

**Affiliations:** 1 Digestive Surgery, Cho Ray Hospital, Ho Chi Minh City, VNM

**Keywords:** colonic surgery, colostomy, diverticulitis, emergencies, perforated sigmoid and rectosigmoid diverticulitis disease, postoperative complications

## Abstract

Background

Colonic diverticula are mucosal pouch anomalies extending through vulnerable areas of the colonic wall. While common in Western nations, managing complicated perforated sigmoid and rectosigmoid diverticulitis remains contentious due to high rates of abscess and peritonitis. Hartmann’s surgery is commonly employed for perforated sigmoid and rectosigmoid diverticulitis due to its efficiency and the mitigation of anastomotic leakage risks. However, reversal remains challenging due to inflammatory adhesions and shortened margins. This study aims to elucidate the morbidity and mortality associated with Hartmann’s operation for perforated sigmoid colon and rectosigmoid diverticula.

Methodology

This retrospective cohort study evaluated 96 patients diagnosed with perforated sigmoid and rectosigmoid diverticulitis who underwent open Hartmann’s procedures at the Department of Digestive Surgery, Cho Ray Hospital, between May 2020 and April 2025. Demographic profiles, surgical characteristics, and 30-day postoperative complications and mortality rates were documented.

Results

The cohort included 50 males and 46 females with a mean age of 67.7 ± 11.4 years; 42 (43.75%) patients were over 70 years of age. Preoperative stratification that showed 60 (62.5%) patients were American Society of Anesthesiologists (ASA) Class II and 36 (37.5%) were ASA Class III. The mean postoperative stay was 7.1 ± 4.8 days, and the overall 30-day postoperative mortality rate was 20.8% (n = 20). Surgical site infections (all superficial incisional) occurred in 24% (n = 23) of cases, and postoperative pneumonia (confirmed by postoperative chest X-ray) occurred in 21.9% (n = 20). Univariate analysis showed that advanced age (>70 years) and intraoperative fecal contamination significantly increased complication risks (p = 0.015 and p = 0.026, respectively). Predictors correlating with increased mortality in univariate analysis included an admission systolic blood pressure (SBP) <90 mmHg (p < 0.001) and fecal peritonitis (p = 0.028). Mortality rates increased with disease severity, reaching 38.1% for Hinchey Grade IV, in contrast to 19.3% for Grade III and 5.6% for Grade II (p = 0.05). Multivariable logistic regression indicated that age exceeding 70 years (odds ratio (OR) = 3.085, p = 0.013) and fecal contamination (OR = 3.328, p = 0.024) were independent predictors of postoperative complications, whereas an admission SBP below 90 mmHg (OR = 16.621, p = 0.001) was the sole independent predictor of mortality.

Conclusions

Hartmann’s procedure is an appropriate intervention for severe perforated sigmoid and rectosigmoid diverticulitis with fecal contamination of the abdominal cavity, shock, or patients with multiple comorbidities. Baseline patient factors heavily dictate survival and morbidity outcomes, with advanced age, intraoperative fecal contamination, and admission hypotension serving as critical prognostic drivers.

## Introduction

Colonic diverticula are saccular anomalies of the mucosa that extend from the colonic lumen through a vulnerable area in the colonic wall. This is a very common disease in Western countries, affecting up to two-thirds of patients over 80 years of age [1,2]. Colonic diverticulitis results from the inflammation of diverticula, affecting around 20-25% of those with a history of diverticula. It can be categorized as simple (uncomplicated) or complex (complicated) [1,3]. In the United States, approximately 12% of patients with colonic diverticulitis encounter problems necessitating hospitalization, including abscess, perforation, colonic fistula, or intestinal blockage, while 20% may face recurrence [4].

The advancement of imaging modalities such as ultrasonography, colonoscopy, and computed tomography has led to earlier and more precise diagnoses of colonic diverticulitis, thus decreasing postoperative complications and death rates. Colonic diverticula may occur throughout the colon, with the sigmoid and rectosigmoid colon being the predominant site, particularly in Western nations. Conversely, right colonic diverticula are more prevalent in Asian countries, including Japan, Korea, and Vietnam.

Most patients with uncomplicated colonic diverticulitis respond well to medical treatment, with a success rate of 95-97% [5-7]. While most right colonic diverticula can be effectively managed medically, sigmoid and rectosigmoid colons with Hinchey III and IV may improve with surgery due to serious intra-abdominal infection, potentially leading to severe complications such as diverticular abscess and peritonitis, rendering treatment approaches contentious. Numerous new clinical guidelines endorse the diagnosis and selection of treatment modalities, including percutaneous drainage of organized diverticular abscesses to minimize the need for surgery at the acute stage, simple diverticulectomy, Hartmann’s surgery, colectomy, and creating an anastomosis or colostomy [6,7]. Nevertheless, problems persist at elevated rates, including surgical wound infection, anastomotic leakage, and postoperative intestinal obstruction, among others. The mortality rate remains constant in hospitalized patients with pre-existing problems of the diverticulum [7].

Hartmann’s surgery is a frequently employed procedure for the management of sigmoid and rectosigmoid colon diverticulitis complicated by perforation. The benefits of Hartmann’s surgery are its expediency, simplicity of execution, and absence of concerns regarding anastomotic leakage problems. Nevertheless, Hartmann’s surgery possesses the same drawbacks associated with colostomy, including prolapse, necrosis, and hernia, which might impact the patient’s psychological well-being. Furthermore, the restoration of intestinal continuity is challenging due to the low position, shortened colon, and inflammatory adhesions following surgery. Notwithstanding these drawbacks, this surgical procedure remains the preferred option for patients with peritonitis resulting from perforation of the sigmoid and rectosigmoid colon diverticula. In 2020, the World Society of Emergency Surgery revised its guidelines for managing acute left colonic diverticulitis, integrating advancements in the treatment of right colonic diverticulitis. The postoperative mortality rate for grade 3 and 4 Hinchey peritonitis was 10.8%, and it is advised to employ Hartmann’s procedure for managing generalized peritonitis in severely sick patients with many comorbidities [7]. This study aimed to elucidate the morbidity and mortality associated with Hartmann’s procedure for perforated sigmoid and rectosigmoid colon diverticulitis.

## Materials and methods

A retrospective cohort study was conducted among 96 patients diagnosed with perforated sigmoid and rectosigmoid colon diverticula who had undergone only an open Hartmann’s procedure at the Department of Digestive Surgery, Cho Ray Hospital, from May 2020 to April 2025. The ethical committee of Cho Ray Hospital approved this study (approval number: 311).

Inclusion and exclusion criteria

Patients who had undergone Hartmann’s open surgery, had a postoperative diagnosis of perforated sigmoid and rectosigmoid colon diverticula, and whose postoperative histopathology findings indicated diverticulitis were considered for inclusion in the study. Patients with insufficient records, data, and images were excluded from the study. Patients with postoperative pathology of diverticulitis with cancer, tuberculosis, and Crohn’s disease were also excluded.

We defined fecal peritonitis as a severe, life-threatening inflammation of the peritoneum caused by fecal matter leaking into the abdominal cavity. Demographic characteristics, surgical characteristics, and postoperative complications and mortality within 30 days were documented. This study documented complications such as surgical site infection, postoperative pneumonia, reoperation, and mortality. Surgical site infections included superficial incisional, deep incisional, and organ/space infections. Postoperative pneumonia was detected on a postoperative chest X-ray.

Management and monitoring

Preoperative diagnoses relied on clinical presentation combined with CT scans showing pneumoperitoneum, extraluminal air, or fluid collections. Hartmann’s surgery was selected based on intraoperative findings of generalized purulent/fecal peritonitis (Hinchey III/IV) or severe hemodynamic instability.

Patients received standard preoperative resuscitation (fluid therapy and antibiotics), including all patients presenting with generalized fecal/purulent peritonitis (Hinchey III/IV), pneumoperitoneum with an acute abdomen, or refractory septic shock. Surgery needed to occur concurrently with rapid volume resuscitation and immediate intervention. Hartmann’s procedures were performed by dedicated colorectal surgeons.

Standardization of operative technique

All operations were performed by senior general surgical teams and followed a standardized protocol using an open approach. Access to the abdomen was achieved by a midline laparotomy incision. Peritoneal fluid was collected at entry for immediate microbiological culture, and grossly purulent or fecal contamination was evacuated. The left colon was mobilized by cutting the white line of Toldt and dividing the phrenicocolic ligament. The ureter and the gonadal vessels were carefully identified and preserved. The diseased portion of the sigmoid was isolated, and the mesenteric vessels were ligated close to the bowel wall with suture ligation or energy devices. Proximal colonic resection margins were through macroscopically healthy tissue without edema. The distal rectal transection was performed at the upper rectum below the promontory with complete clearance of the perforated area. The distal rectal stump was closed with a linear stapler (60 mm) or a two-layer hand-sewn technique with 3-0 polyglactin sutures. Systematic extensive intraoperative peritoneal lavage with 3 to 5 L of warm normal saline was performed until the effluent was crystal clear. A standard left lower quadrant terminal end-colostomy was matured through a separate circular skin incision with interrupted 3-0 absorbable sutures. After anatomical abdominal closure, one or two Silastic drains were left in the pelvis and the paracolic gutter for abdominal drainage.

Statistical analysis

Data were processed and analyzed using SPSS version 20.0 software (IBM Corp., Armonk, NY, USA). Categorical variables were evaluated using the chi-square test or Fisher’s exact test where expected frequencies were less than 5. Continuous variables were assessed using the independent Student’s t-test or the Mann-Whitney U-test based on normal distributions. Independent risk factors for postoperative complications and 30-day mortality were determined using multivariable logistic regression. Rather than relying strictly on univariable screening, variables were selected a priori based on clinically established prognostic markers for diverticular disease, including advanced age, preoperative hypotension, and intraoperative fecal contamination. A backward elimination strategy was utilized to optimize final model fit, reporting adjusted odds ratios (ORs) with 95% confidence intervals (CIs). Missing data were handled via listwise deletion; however, complete clinical records were available for all final model components. Statistical significance was established at a p-value <0.05.

## Results

The study comprised 50 (52.1%) males and 46 (47.9%) females, resulting in a male-to-female ratio of 1.08:1. The mean age in the study was 67.7 ± 11.4 years (ranging from 31 to 92 years), with 42 (43.75%) participants being over 70 years of age. The study included 96 patients, with 60 (62.5%) classified as American Society of Anesthesiologists (ASA) II and 36 (37.5%) as ASA III. The mean body mass index was 22.4 ± 2.5 kg/m², and 24 (25.0%) patients had septic shock. The mean operative time was 150 ± 25 minutes, and intraoperative blood loss was not documented. The predominant clinical manifestation was abdominal discomfort in the right lower quadrant, left lower quadrant, hypogastric area, and across the abdomen. The mean duration from the onset of stomach pain to admission was three days (range = 1-5 days). The initial symptoms comprised abdominal discomfort, nausea or vomiting, diarrhea, fever, abdominal distension, and vital signs, including SBP, recorded at the time of admission. Comorbidities were categorized into the following groups: diabetes mellitus, cardiovascular disease, and chronic obstructive pulmonary disease. Hinchey classification utilized during surgery is elaborated on in Table 1. The mean postoperative time was 7.1 ± 4.8 days (range = 1-32).

**Table 1:** Demographic characteristics and clinical symptoms. COPD: chronic obstructive pulmonary disease; SBP: systolic blood pressure; SD: standard deviation

Sex	Male	Female	Total
	50 (52.1%)	46 (47.9%)	96 (100%)
Age (years) (mean ± SD)	67.8 ± 11.2	67.6 ± 8	67.7 ± 11.4
Location of abdominal pain	n = 50	n = 46	n = 96
Left lower quadrant	22	15	37 (38.5%)
Right lower quadrant	2	0	2 (2.1%)
Lower abdomen	16	20	36 (37.5%)
Throughout the abdomen	10	11	21 (31.9%)
Comorbidities, n = 96 (100%)			
Diabetes	26	27.08%	
Cardiovascular disease	53	55.02%	
COPD	10	10.41%	
Symptom, n = 96 (100%)			
Nausea or vomiting	15	15.6%	
Diarrhea	8	8.3%	
SBP <90 mmHg	24	25%	
Fever ≥38°C	28	29.2%	
Abdominal bloating	76	79.2%	
Resistance	91	94.8%	
Hinchey grade, n = 96 (100%)			
II	18	18.8%	
III	57	59.3%	
IV	21	21.9%	
Complications and mortality, n = 96 (100%)			
Postoperative pneumonia	21	21.9	
Surgery site infection (superficial incisional)	23	24	
Mortality	20	20.8	

Abdominal resistance and distension were the two predominant symptoms, occurring in 91 (94.8%) and 76 (79.2%) cases, respectively. In 24 (25.0%) cases, SBP was below 90 mmHg, and fever was present in 28 (29.2%) cases. However, when SBP fell below 90 mmHg, the mortality risk was 12 of 24 (50.0%), accounting for 12 of 20 (60.0%) patient deaths (p < 0.001). Table 2 provides a comprehensive description of the correlation among clinical symptoms, complications, and mortality.

**Table 2:** Comparison of clinical characteristics with complications and mortality. SBP: systolic blood pressure

Factor	Total patients (n = 96)	Complication (n = 35)	Mortality (n = 20)	P-value
Nausea or vomiting	15 (15.6%)	4 (11.4%)	2 (10%)	0.730
Diarrhea	8 (8.3%)	3 (8.6%)	1 (5%)	1.000
SBP <90 mmHg	24 (25%)	11 (31.4%)	12 (60%)	<0.001
Fever ≥38°C	28 (29.2%)	13 (37.1%)	6 (30%)	0.193
Abdominal bloating	76 (79.2%)	27 (77.1%)	16 (80%)	0.056
Resistance	91 (94.8%)	35 (100%)	19 (95%)	0.155

The majority of patients were admitted with generalized peritonitis, comprising 57 (59.3%) patients with purulent peritonitis (Hinchey grade 3) and 21 (21.9%) patients with fecal peritonitis (Hinchey grade 4). There were only 18 (18.8%) cases of a pelvic abscess (Hinchey grade 2). Instances of fecal peritonitis (Hinchey grade 4) exhibited a markedly elevated incidence of postoperative pneumonia at 9 (42.9%) in comparison to other grades (Fisher’s test, p < 0.05). The incidence of surgical site infections in the fecal peritonitis cohort was 6 (28.6%), exceeding that of the other groups. This difference was not statistically significant (chi-square test, p > 0.05). The Hinchey grade 4 group exhibited the highest mortality rate at 8 (38.1%), followed by grades 3 and 2 with rates of 11 (19.3%) and 1 (5.6%), respectively. The difference was borderline statistically significant with a p-value of 0.05 (Fisher’s test) (Table 3).

**Table 3:** Hinchey grade and its correlation with complications and mortality.

Hinchey grade	II	III	IV	P-value
Postoperative pneumonia	2 (11.1%)	10 (17.5%)	9 (42.9%)	0.036
Surgery site infection (superficial incisional)	5 (27.8%)	12 (21.1%)	6 (28.6%)	0.721
Mortality	1 (5.6%)	11 (19.3%)	8 (38.1%)	0.05

The most frequent problem was superficial incisional surgical site infection in 23 (24.0%) patients, and the overall postoperative mortality rate was 20.8% (20 cases). Univariate analysis showed that age >70 years and intraoperative fecal debris were significantly associated with the overall composite endpoint of postoperative complications (surgical site infection, pneumonia, reoperation, and mortality). In particular, among the 35 complicated cases, 21 (60.0%) patients were >70 years old, and 12 (34.3%) had intraoperative fecal contamination. When each complication was considered individually, age >70 years was associated with an increased risk of postoperative pneumonia, which occurred in 21 patients (50.0% of that age cohort, p = 0.015). On the other hand, intraoperative fecal matter was linked to a significant increase in the risk of overall complications to 57.1% (12 cases; p = 0.026) (Table 4).

**Table 4:** Univariate analysis of factors associated with complications. SBP: systolic blood pressure

Factor		Complication		P-value
		Yes	No	
Sex	Male	22 (44%)	28 (56%)	0.109
	Female	13 (28.3%)	33 (71.7%)	
Age (years)	≤70	14 (25.9%)	40 (74.1%)	0.015
	>70	21 (50%)	21 (50%)	
SBP (mmHg)	≥ 90	24 (33.3%)	48 (66.6%)	0.271
	<90	11 (45.8%)	13 (54.2%)	
Comorbidities	Yes	22 (34.9%)	41 (65.1%)	0.665
	No	13 (39.4%)	20 (60.6%)	
Feces in the abdominal cavity	Yes	12 (57.1%)	9 (42.9%)	0.026
	No	23 (30.7%)	52 (69.3%)	

Among the 20 patients who succumbed post-surgery, 12 (60%) exhibited hypotension (SBP <90 mmHg) due to preoperative septic shock. During the surgical procedure, 8 (40%) patients exhibited the presence of fecal matter within the abdominal cavity. Postoperatively, 13 (65%) patients had pneumonia. Factors associated with postoperative mortality included SBP below 90 mmHg at admission, the presence of fecal matter in the abdominal cavity, and postoperative pneumonia. Factors elevating the postoperative mortality rate encompassed SBP <90 mmHg at admission, with a 50% mortality risk (p < 0.001); the presence of fecal matter in the abdominal cavity during surgery, associated with a 38.1% mortality risk (p = 0.028); and postoperative pneumonia, which carried a mortality risk exceeding 60% (p < 0.001) (Table 5).

**Table 5:** Univariate analysis of factors associated with mortality. SBP: systolic blood pressure

Factor		Mortality		P-value
		Yes	No	
Sex	Male	12 (24%)	38 (76%)	0.426
	Female	8 (17.4%)	38 (82.6%)	
Age (years)	≤70	10 (18.5%)	44 (81.5%)	0.527
	>70	10 (23.8%)	32 (76.2%)	
SBP (mmHg)	≥ 90	8 (11.1%)	64 (88.9%)	<0.001
	<90	12 (50%)	12 (50%)	
Comorbidities	Yes	12 (19%)	51 (81%)	0.552
	No	8 (24.2%)	25 (75.8%)	
Feces in the abdominal cavity	Yes	8 (38.1%)	13 (61.9%)	0.028
	No	12 (16%)	63 (84%)	
Postoperative pneumonia	Yes	13 (61.9%)	8 (38.1%)	<0.001
	No	7 (9.3%)	68 (90.7%)	

A multivariate logistic regression analysis was conducted to ascertain independent prognostic markers. The findings indicated that age over 70 years and the presence of stool in the abdominal cavity during surgery were two independent predictive variables for postoperative pneumonia and surgical site infection. Likewise, an SBP at admission of less than 90 mmHg was an independent predictor of mortality (Table 6). We did not have readmissions and reinterventions (including percutaneous drainage and returns to the theater) captured within 30 days post-discharge.

**Table 6:** Multivariate analysis of factors associated with postoperative complications and mortality. CI: confidence interval; OR: odds ratio; SBP: systolic blood pressure

	Factor	OR	95% CI	P-value
Complication	Age >70 years	3.085	1.64–7.533	0.013
	Feces in the abdominal cavity	3.328	1.175–9.422	0.024
Mortality	SBP <90 mmHg	16.621	3.207–86.140	0.001
	Feces in the abdominal cavity	1.191	0.297–4.769	0.805

## Discussion

Colon diverticulitis exacerbated by perforation is an increasingly prevalent condition among older individuals, both in developed and developing nations. This study examined patients with perforated sigmoid and rectosigmoid colon diverticula who underwent emergency Hartmann’s surgery to elucidate surgical results, postoperative complications, and mortality rates within this cohort.

The mean age of the participants in the study was 67.7 ± 11.4 years, consistent with findings from prior studies [8,9]. Physiological factors of aging have been identified as playing a central role in the pathogenesis of colon diverticulitis, including decreased bowel motility, decreased bowel wall muscle tone, changes in the gut microbiota, increased intraluminal pressure, and immunosuppression. Our study exhibited the largest proportion of individuals aged over 70 years at 43.8%, surpassing Oh et al.’s figure of 23.4%. This discrepancy may arise from the increased prevalence of sigmoid and rectosigmoid diverticula perforation among older adults [10]. The study had 50 male participants (52.1%) and 46 female participants (47.9%), with a male-to-female ratio of 1.08:1. The age and sex distributions were generally comparable to those reported in previous studies [8,9].

All patients in our study cohort underwent open surgery because most were emergency scenarios characterized by abdominal distension, infection, inadequate bowel preparation, and the patient’s overall condition. Laparoscopic surgery is the prevailing global trend, offering benefits over open surgery, including diminished pain, lower incidence of wound infection, expedited recovery, reduced hospital stays, and comparable mortality, complication, and reoperation rates within 30 days, even among elderly patients (>75 years old) [11]. Laparoscopic surgery for pelvic and rectal diverticulitis remains technically challenging due to emergency conditions, constrained surgical fields, bowel distension, and severe abdominal contamination. Despite these complexities, current literature indicates that laparoscopic surgery for sigmoid colon resection is a safe, practical, and highly effective alternative to open surgery across all stages of diverticulitis, including Hinchey III-IV peritonitis [12-15]. Studies demonstrate that this minimally invasive approach yields low overall complication and mortality rates, reduced reoperation frequencies, shorter hospital stays, and expedited patient recovery, positioning it as a modern standard of care [12-15]. The drawback of our study is that we exclusively selected cases with open Hartmann’s surgery, precluding evaluation and comparison with laparoscopic surgery.

In cases of sigmoid and rectosigmoid diverticulitis complicated by abscess or widespread peritonitis, the preferred surgical approach typically involves excision of the colon harboring the diverticulum, formation of a stoma, or immediate anastomosis, contingent upon the individual patient’s condition [16,17]. In our study, we admitted the majority of patients with peritonitis (81.3%). We mostly opted for open surgery from the beginning, involving the removal of the pelvic colon and the establishment of a colostomy, eschewing colon anastomosis in the context of peritonitis, which elevates the risk of leakage. The selection of surgical techniques remains contentious among authors globally.

The selection of colon resection and anastomosis from the beginning, with or without the formation of a colostomy, is progressively supplanting Hartmann’s procedure in the management of colonic diverticulitis and is increasingly prevalent in the treatment of peritonitis resulting from sigmoid and rectosigmoid diverticula, extending beyond mere instances of colonic diverticulitis. The primary advantage of this procedure is the mitigation of risk for patients during the closure of the colostomy in the second stage, thus decreasing expenditures and shortening the duration of hospital stays [16]. Abbas indicated that the incidence of anastomotic leakage following the closure of Hartmann’s colostomy exceeds that of prompt closure in the initial stage (8% > 5.5%) [17]. In our analysis, the majority of senior patients, averaging 67.7 years, were late with peritonitis, comorbidities, and septic shock, prompting the surgeon to typically select Hartmann’s procedure as the right intervention. While Hartmann’s procedure has historically been the standard intervention for perforated left colonic diverticulitis, contemporary surgical paradigms and guidelines increasingly advocate for primary resection and anastomosis, with or without a diverting loop ileostomy, in hemodynamically stable patients [18,19]. However, because our study exclusively evaluated open Hartmann’s procedures within a retrospective, single-arm framework, we cannot determine or imply the clinical superiority of Hartmann’s procedure over alternative strategies such as primary resection and anastomosis or laparoscopic lavage. Instead, our data confirm that Hartmann’s procedure remains a pragmatic, rapid choice for rapid abdominal sepsis control in highly unstable patients where performing an anastomosis is unsafe.

Patients necessitating emergency surgery for acute diverticulitis with complicated perforation were frequently managed with Hartmann surgery; however, recently, colectomy and immediate anastomosis, with or without suprastomal colostomy and laparoscopic lavage, have gained prevalence and are more broadly utilized for patients with purulent peritonitis. Nonetheless, there is a lack of credible evidence from randomized trials about these treatments, and studies indicating favorable outcomes are attributed to meticulously selected patient cohorts [20]. Hartmann’s procedure remains the predominant emergency intervention for complicated diverticulitis, accounting for 72% of emergency colectomy cases [21]. It is strongly indicated in acute scenarios complicated by hemodynamic instability, regardless of peritonitis severity, and encompasses both purulent (Hinchey III) and fecal (Hinchey IV) peritonitis [22].

The mean duration of postoperative stay in the study was 7.1 days, ranging from a minimum of 1 day to a maximum of 32 days. Overall, 35 patients experienced one or more problems, representing 36.5% of the sample, which is comparable to the findings of 14.9% to 33.4% in other studies [8,9,17,23]. Advanced age, obesity, preoperative comorbidities (such as diabetes, liver disease, and hypertension), and extended corticosteroid usage influence complications [24]. Prior literature highlights the critical impact of baseline patient health on postoperative outcomes following diverticulitis surgery. Specifically, Takano et al. demonstrated that the presence or absence of comorbidities directly influences the incidence of targeted postoperative complications, most notably surgical site infections and postoperative pneumonia [25]. Aligning the focus to these specific variables underscores how pre-existing medical conditions dictate immediate postoperative recovery trajectories. Consequently, for patients with a history of diverticulitis, the evaluation of a therapeutic strategy remains contentious. Historically, routine sigmoid colectomy was warranted for diverticulitis in cases of immunosuppression, recurrence exceeding two episodes, complicated diverticulitis, drained diverticulitis abscess, luminal stricture, and gastrointestinal fistula. Conversely, emergency surgery remained necessary for instances of unsuccessful medical management of complicated diverticulitis, peritonitis, multiple abscesses, inability to drain or ineffective drainage, and bowel obstruction unresponsive to medical treatment [26].

Surgical site infection was the most common complication after surgery, affecting 24% of patients. The incidence of surgical site infection in our study was comparable to findings from international studies, varying between 4.6% and 34% of cases [23,25,27]. Conditions such as diabetes, malnutrition, extended surgical duration, inadequate surgical hygiene, lengthy incisions, or immunosuppression heighten the likelihood of surgical site infection. Schietroma et al.’s recent perspective [27] indicates that oxygen tension in tissues is frequently diminished at the surgical site and during colon anastomosis, impeding the healing process by hindering the oxidative function of phagocytosis by polymorphonuclear leukocytes, diminishing collagen synthesis, and enhancing vascular and epithelial proliferation. The study proposed that administering hyperbaric oxygen (FiO₂ > 80) during surgery and for six hours postoperatively may diminish the incidence of surgical site infections and anastomotic leaks following surgery for colonic diverticulitis. Our investigation documented respiratory problems, including postoperative pneumonia in 21 (21.9%) cases. This rate exceeds that documented by other studies, which ranged from 1% to 15.7% of cases [9,23,25].

The mortality rate linked to pneumonia post-surgery ranges from 20% to 50%, contingent upon the surgical procedure, and adversely affects early postoperative recovery and the quality of life of patients. Postoperative pneumonia is the second most severe complication after surgical site infection and poses a significant risk following surgery for colonic diverticulitis. Recent research indicates that postoperative pneumonia following gastrointestinal surgery not only prolongs hospital stays but is also independently linked to an elevated mortality risk [28]. Postoperative pneumonia during emergency Hartmann’s surgery results from the interplay of patient and surgical factors. Patients aged over 70 years and those with feces present during surgery were independent prognostic variables linked to postoperative pneumonia and surgical site infection. Advanced age, chronic pulmonary disease, inadequate nutritional status, and preoperative infection are variables that elevate risk. Patients undergoing emergency surgery for peritonitis, with an unprepared colon, protracted intubation, resuscitation, and extended mechanical ventilation, are at an elevated risk of postoperative pneumonia. Xiang et al.’s study demonstrated that extended mechanical ventilation duration (>24 hours) is an independent factor linked to a heightened risk of postoperative pneumonia [29].

While international literature reports post-diverticulitis reoperation rates ranging widely from 4.1% to 36.2%, our cohort required no surgical reinterventions [23,24]. This low rate is directly reflected in our favorable postoperative outcomes; specifically, no patients experienced major complications that typically trigger reoperation, such as abdominal wall rupture, residual abscesses, early bowel obstruction, or stoma-related issues (including colonic anal prolapse, blockage, necrosis, or hernia). The total mortality in the study was 20 cases, representing 20.8% of the study sample. This percentage surpasses those of other studies, which range from 0.19% to 19% of cases [7,12,14,16,17,23,24]. Our study sample comprised cases of pelvic and rectal diverticulitis complicated by perforation, involving older patients with various comorbidities necessitating immediate surgical intervention due to peritonitis. Diamant et al. also reported that the mortality rate was higher for women, people over 60 years of age, those with serious comorbidities, those who were admitted late, those who were in an emergency situation, or those who had surgery [30].

The mortality rate was also higher for people who were admitted to smaller, non-specialized hospitals than for people who were admitted to larger medical facilities. In cases of colonic diverticulitis with perforation complications, the mortality rate for uncomplicated diverticulitis is approximately 0.1%. However, this rate escalates to 4% in the presence of peritonitis and surges to 32% when accompanied by organ failure. Moreover, immunocompromised individuals, organ transplant recipients, those with organ failure, and patients with end-stage renal failure exhibit markedly elevated mortality rates. Notwithstanding significant progress in early diagnosis, surgical methodologies, antibiotics, and postoperative resuscitation, the mortality rate resulting from the perforation of pelvic and upper rectal diverticula remains very high, exhibiting minimal decline. Our investigation identified older patients (>70 years), those with an SBP <90 mmHg upon admission, stool presentation during surgery, and postoperative pneumonia as risk factors for mortality. Among all analyzed variables, preoperative hypotension (admission SBP <90 mmHg) emerged as the single most critical independent predictor of 30-day mortality, multiplying the risk of death more than sixteen-fold (OR = 16.621, p = 0.001). This massive statistical significance shifts the clinical focus from intraoperative technical factors to the early preoperative resuscitation window. It demonstrates that patient survival is ultimately dictated by systemic hypoperfusion and early multi-organ failure rather than the extent of local contamination alone, demanding hyper-aggressive fluid and vasopressor resuscitation within the first hours of admission.

Study limitations

This is a retrospective study involving a relatively small number of patients at a single center and does not compare Hartmann’s surgery with other surgical procedures. The follow-up period was short at only 30 days post-surgery. Other relevant outcomes (e.g., intra-abdominal abscesses, prolonged ileus, acute kidney injury, or stoma complications) were not captured; this data collection boundary is a limitation. Only open surgery was performed, and the role of laparoscopic surgery was not evaluated. These limitations provide a basis for future prospective or controlled studies to provide stronger evidence for choosing the optimal surgical procedure for patients.

## Conclusions

Our findings show that Hartmann’s procedure is still an important emergency option for the treatment of severe perforated sigmoid colon and rectal diverticula under true damage-control conditions. Because of the retrospective nature of our study and the absence of a concurrent comparative cohort, these data cannot demonstrate the superiority of Hartmann’s procedure over other surgical options, such as primary resection with anastomosis. Instead, our results highlight that the prognostic influence of advanced age (>70 years), intraoperative fecal contamination, and admission hypotension (SBP <90 mmHg) is heavily detrimental to postoperative success. Furthermore, the long-term success of the procedure is unquantified, as this study was confined to the 30-day postoperative window and lacked long-term survival metrics and stoma reversal outcomes. Future prospective, comparative trials are needed to optimize patient selection and refine algorithms for emergency management.
